# Integrative single-cell RNA-seq and spatial transcriptomics analyses reveal diverse apoptosis-related gene expression profiles in *EGFR*-mutated lung cancer

**DOI:** 10.1038/s41419-024-06940-y

**Published:** 2024-08-09

**Authors:** Motohiro Izumi, Masanori Fujii, Ikei S. Kobayashi, Vivian Ho, Yukie Kashima, Hibiki Udagawa, Daniel B. Costa, Susumu S. Kobayashi

**Affiliations:** 1 Department of Medicine, Division of Medical Oncology, Beth Israel Deaconess Medical Center, Harvard Medical School, Boston, MA 02215 USA; 2 Division of Translational Genomics, Exploratory Oncology Research and Clinical Trial Center, National Cancer Center, Kashiwa, 277-8577 Japan; 3 Department of Thoracic Oncology, National Cancer Center Hospital East, Kashiwa, 277-8577 Japan; 4 Department of Respiratory Medicine, Juntendo University Faculty of Medicine and Graduate School of Medicine, Tokyo, 113-8431 Japan

**Keywords:** Non-small-cell lung cancer, Transcriptomics

## Abstract

In *EGFR*-mutated lung cancer, the duration of response to tyrosine kinase inhibitors (TKIs) is limited by the development of acquired drug resistance. Despite the crucial role played by apoptosis-related genes in tumor cell survival, how their expression changes as resistance to EGFR-TKIs emerges remains unclear. Here, we conduct a comprehensive analysis of apoptosis-related genes, including BCL-2 and IAP family members, using single-cell RNA sequence **(**scRNA-seq) and spatial transcriptomics (ST). scRNA-seq of *EGFR*-mutated lung cancer cell lines captures changes in apoptosis-related gene expression following EGFR-TKI treatment, most notably *BCL2L1* upregulation. scRNA-seq of *EGFR*-mutated lung cancer patient samples also reveals high *BCL2L1* expression, specifically in tumor cells, while *MCL1* expression is lower in tumors compared to non-tumor cells. ST analysis of specimens from transgenic mice with EGFR-driven lung cancer indicates spatial heterogeneity of tumors and corroborates scRNA-seq findings. Genetic ablation and pharmacological inhibition of *BCL2L1*/BCL-XL overcome or delay EGFR-TKI resistance. Overall, our findings indicate that *BCL2L1*/BCL-XL expression is important for tumor cell survival as EGFR-TKI resistance emerges.

## Introduction

Despite improvements in therapeutics and advancements in research, lung cancer remains a leading cause of cancer-related deaths [1]. The discovery of mutations in the *epidermal growth factor receptor* (*EGFR*) gene allowed for the development of tyrosine kinase inhibitors (TKIs) as first-line treatment for advanced/metastatic non-small-cell lung cancer harboring *EGFR* mutations. Treatment with EGFR-TKIs results in a rapid response and improves both progression-free and overall survival [2–4]. Despite these responses, acquired resistance to EGFR-TKIs invariably develops. Such limitations of EGFR-TKI monotherapy prompted us to investigate mechanisms underlying drug resistance and tolerance. It is reported that tumor cells transiently maintain a drug-tolerant state following treatment with targeted therapies, allowing tumor cell subpopulations to withstand and adapt to those drugs [5–7]. This subpopulation, called drug-tolerant persister (DTP) cells, exhibits a relatively slow cell cycle and survives during drug treatment. These characteristics are reversible once the drug is withdrawn. Currently, there is no standardized protocol to establish DTP pre-clinical models [8].

Evasion of apoptotic cell death is a well-known phenomenon in tumor cells, one that leads to drug resistance [7]. A common molecular mechanism used by tumor cells to achieve drug resistance is the overexpression of anti-apoptotic proteins and downregulation of pro-apoptotic proteins [9]. Therefore, we focused on apoptosis-related genes to define mechanisms of EGFR-TKI resistance. The B Cell Lymphoma 2 (BCL-2) family includes both pro-apoptotic (BAX, BAK) and anti-apoptotic (BCL-2, BCL-XL, BCL-w, MCL1) members. Their balanced activity largely governs cell fate decisions between cell survival and death [10]. The inhibitor of apoptosis protein (IAP) family, which consists of eight members (NAIP, BIRC2, BIRC3, XIAP, BIRC5, BIRC6, BIRC7, and BIRC8), is also highly expressed in a variety of human malignancies [11]. Recent studies suggest that anti-apoptotic molecules such as BCL-XL and MCL1 may function in DTP formation in *EGFR*-mutated lung cancer [12–16]. However, many previous studies relied primarily on cell line models, potentially overlooking the impact of tumor heterogeneity and the tumor microenvironment.

Currently, single-cell RNA sequence (scRNA-seq) technologies can assess gene expression at single-cell resolution and provide extensive insight into the cellular composition of tumors [17, 18]. Although lack of a spatial context of cells is a challenge for scRNA-seq analysis, recent advances in spatial transcriptomics (ST) have enabled elucidation of spatial heterogeneity and the tumor microenvironment [19], and are revolutionizing our understanding of cancer biology. However, changes in expression profiles of apoptosis-related genes occurring as resistance to EGFR-TKI emerges have not been evaluated using such multi-omic approaches.

In this study, we perform scRNA-seq to analyze gene expression profiles, including those of apoptosis-related genes, and their response to treatment in *EGFR*-mutated lung cancer using human clinical samples and cell lines and also perform ST analyses using transgenic mouse specimens. We find that *BCL2L1*, which encodes the anti-apoptotic protein BCL-XL, is highly expressed predominantly in tumor cells, where it likely plays a crucial role in tumor cell survival as drug tolerance and acquired resistance to EGFR-TKIs emerge. We also demonstrate the efficacy of targeting BCL-XL in combination with EGFR-TKI treatment to prevent resistance, not only in cell lines but in transgenic mice, which better reflect characteristics of tumors and the tumor microenvironment, as well as anti-tumor immune responses.

## Results

### *BCL2L1* promotes survival of DTPs and drug-resistant cells after osimertinib treatment in cell line models

**Fig. 1:**
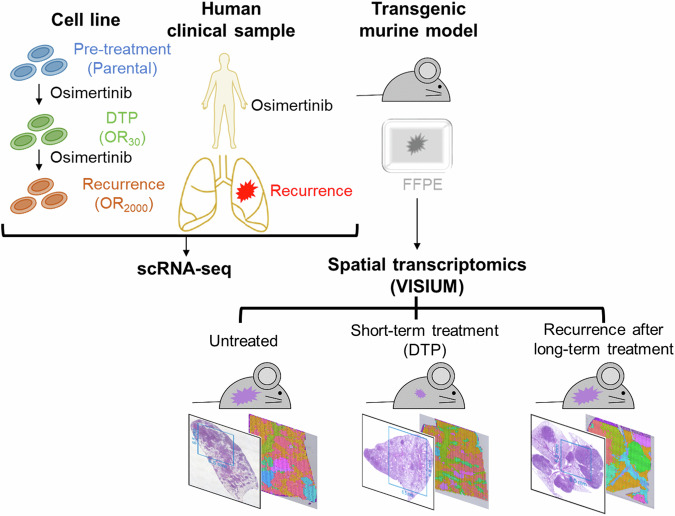
Overview of study. To assess apoptosis-related gene expression in tumors following EGFR-TKI treatment, we established cell line models (top, left) representing untreated, drug-tolerant persister (DTP), and acquired resistance states using *EGFR*-mutated lung cancer lines and subjected them to single-cell RNA sequencing (scRNA-seq). To validate those results and compare gene expression profiles between tumor and normal cells, we conducted scRNA-seq on samples collected from patients with *EGFR*-mutated lung cancer pre-treatment or after the development of EGFR-TKI resistance (top, middle). To confirm scRNA-seq findings and assess spatial heterogeneity, we prepared formalin-fixed paraffin-embedded (FFPE) lung tissue sections from transgenic mice (top, right) harboring EGFR-driven lung cancer representative of three distinct states: untreated, short-term drug treatment reflecting the DTP state, and tumor recurrence after long-term treatment. Spatial transcriptomics was subsequently conducted.

**Fig. 2:**
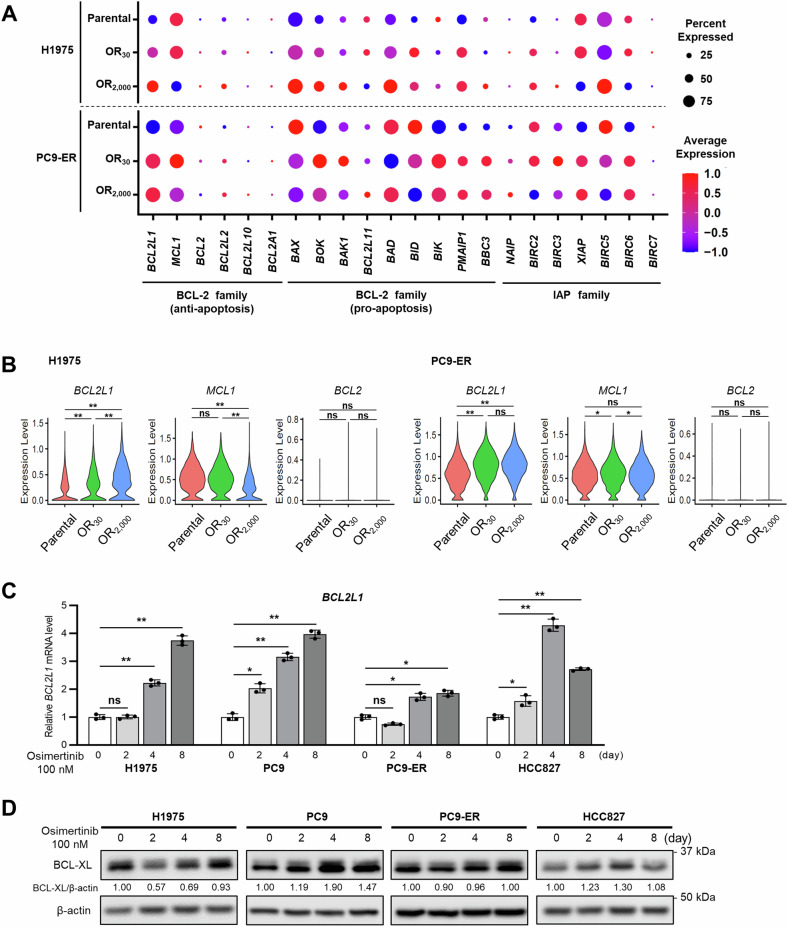
Osimertinib induces *BCL2L1* expression in *EGFR*-mutated lung cancer cell lines. **A** Dotplots based on scRNA-seq showing apoptosis-related gene expression in H1975 (top) and PC9-ER (bottom) cells. **B** Violin plots based on scRNA-seq showing *BCL2L1*, *MCL1*, and *BCL-2* expression in H1975 and PC9-ER cells. *p*-values were calculated using Kruskal–Wallis one-way ANOVA. **C** Relative *BCL2L1* transcript levels in indicated *EGFR*-mutant cell lines based on real-time PCR. Each line was treated with 100 nM osimertinib and evaluated at 0, 2, 4, and 8 days. Data are means ± SD of triplicates from one experiment and are representative of three independent experiments. A Repeated measures one-way ANOVA with Dunnett’s test was used to compare treatment groups to a single control group. **D** Immunoblot analysis showing changes in BCL-XL expression in *EGFR*-mutant lung cancer lines treated with 100 nM osimertinib for 0, 2, 4, and 8 days. The BCL-XL/actin ratio was calculated using ImageJ software. All blots show representative images of three independent experiments. Asterisks indicate *p*-values as follows: *p < 0.05; **p < 0.005; ns, not significant. Data are presented as the mean ± SD.

To investigate cellular responses underlying the acquisition of osimertinib resistance, we used scRNA-seq data from polyclonal osimertinib-resistant cells that we previously generated using H1975 or PC9-erlotinib-resistant (ER) lines [15]. Specifically, we analyzed the expression of apoptosis-related genes in parental lines, OR_30_ cells, which survive in the presence of 30 nM osimertinib, and OR_2000_ cells, which are resistant to 2000 nM osimertinib, as respective models of untreated, DTP, or tumor-recurrent states (see Fig. 1 for study workflow) [15]. We observed significantly higher *BCL2L1* expression in OR_30_ and OR_2000_ cells than in parental cells, while *MCL1* expression was comparable in parental cells and OR_30_ cells and decreased in OR_2000_ cells in both lines (Fig. 2A, B and Supplementary Fig. 1A, B). Expression of other apoptosis-related genes of the BCL-2 and IAP families was either undetectable in >75% of cells or showed no consistent trend in the cell lines used (Fig. 2A, B, Supplementary Fig. 2A–C and 3A–C). This analysis suggests that increased *BCL2L1* expression is a predominant change seen as cells acquire EGFR-TKI resistance.

To validate scRNA-seq results in the DTP state, we confirmed changes in mRNA and protein levels of anti-apoptotic genes after treatment with osimertinib using four *EGFR*-mutated cell lines. When cells were treated with 100 nM osimertinib, a concentration higher than IC_50_s [20], *BCL2L1* mRNA levels significantly increased over time after osimertinib treatment in all lines tested (Fig. 2C). BCL-XL protein levels increased gradually after EGFR-TKI treatment or decreased 2 days after the start of EGFR-TKI treatment and then generally increased over 8 days of treatment (Fig. 2D). These results suggest that BCL-XL may be important for tumor cell survival during emergence of drug tolerance and development of acquired resistance to EGFR-TKIs.

### *BCL2L1* is specifically highly expressed in tumors in clinical samples

**Fig. 3:**
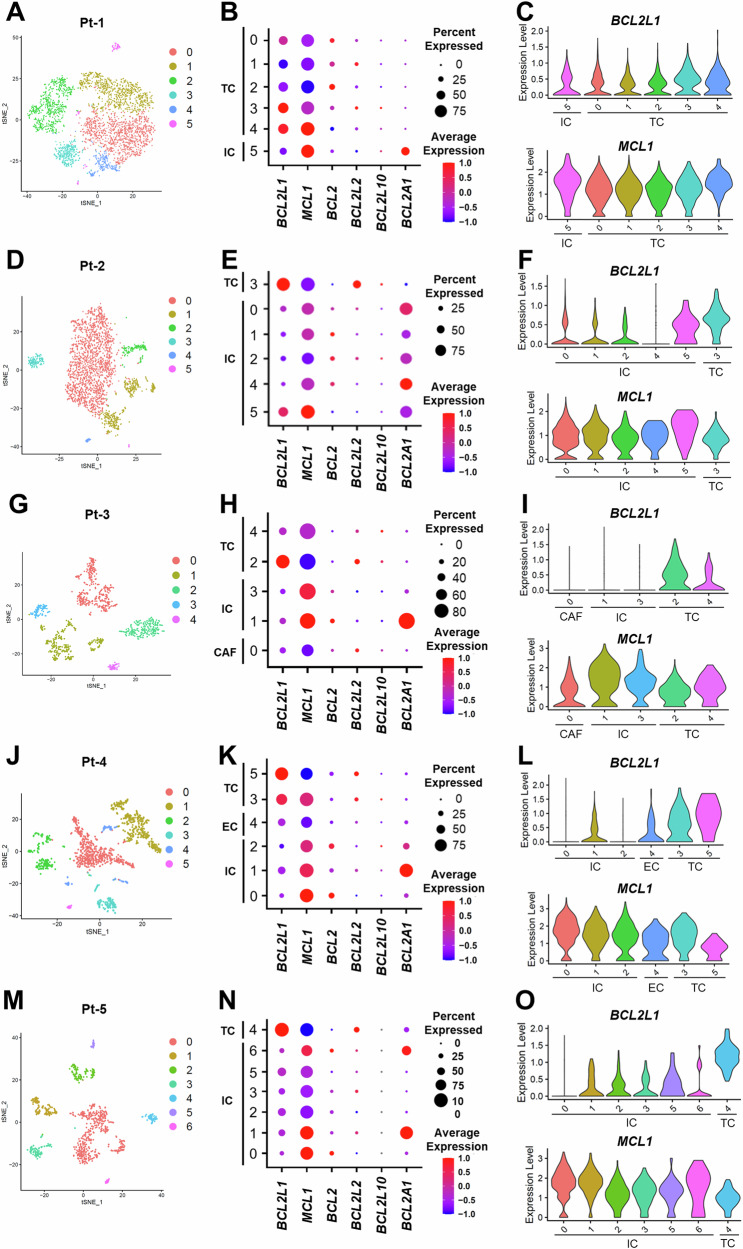
Apoptosis-related gene expression in clinical samples. **A**, **D**, **G**, **J**, **M** t-SNE plots showing clusters based on scRNA-seq in indicated patients. **B**, **E**, **H**, **K**, **N** Dot plots showing differences in expression of anti-apoptotic genes in tumor versus non-tumor cells. **C**, **F**, **I**, **L**, **O** Violin plots showing *BCL2L1* (upper) and *MCL1* (lower) expression in indicated cell types, indicating that *BCL2L1* is specifically and highly expressed in tumor cells. TC tumor cell, IC immune cell, EC endothelial cell, CAF cancer-associated fibroblast.

Next, we analyzed scRNA-seq data from patients with *EGFR*-mutated lung cancer before EGFR-TKI treatment and after the development of drug resistance to confirm results seen in cell lines and compare gene expression levels in tumor and non-tumor cells (Fig. 1). To do so, we analyzed scRNA-seq data from five clinical samples: two osimertinib failure cases (Pt-1 and Pt-4), two first- or second-generation EGFR-TKI failure cases (Pt-2 and Pt-3), and one untreated case (Pt-5). Characteristics of Pt-1, Pt-2, Pt-3, and Pt-4 have been previously reported [15], while characteristics of Pt-5 are shown in Supplementary Table 1. Clusters were annotated based on expression of representative marker genes, as previously reported [15]: those included *EPCAM* for epithelial cells, *PTPRC* for CD45-positive immune cells, *VCAM1* for cancer-associated fibroblasts, and *VWF* for endothelial cells. We employed inferCNV to predict copy number variations to identify distinct copy number profiles in *EPCAM*-positive tumor cells compared to non-tumor cells (Supplementary Fig. 4A–C and 5A, B). In all cases, a high percentage of tumor cells expressed *BCL2L1* and *MCL1* as anti-apoptotic genes of the BCL-2 family (Fig. 3B, E, H, K, N). However, *BCL2L1* was highly expressed specifically in tumor cells, while *MCL1* was expressed at lower levels in tumors compared to non-tumor cells (Fig. 3A–O). Among pro-apoptotic BCL-2 family genes, *BID* expression was lower in tumor cells, and in the IAP family, *XIAP* expression tended to be higher in tumors relative to non-tumor cells (Supplementary Fig. 6). To validate our findings, we analyzed another scRNA-seq dataset (GSE146100) [21] from lung adenocarcinoma patients. We consistently found that *BCL2L1* and *XIAP* were highly expressed specifically in tumor cells but not in non-tumor cells. Conversely, *MCL1* and *BID* expression were lower in tumor cells compared to non-tumor cells. (Supplementary Fig. 7A–E).

### Identification of inter- and intra-tumor heterogeneity using spatial transcriptomics

**Fig. 4:**
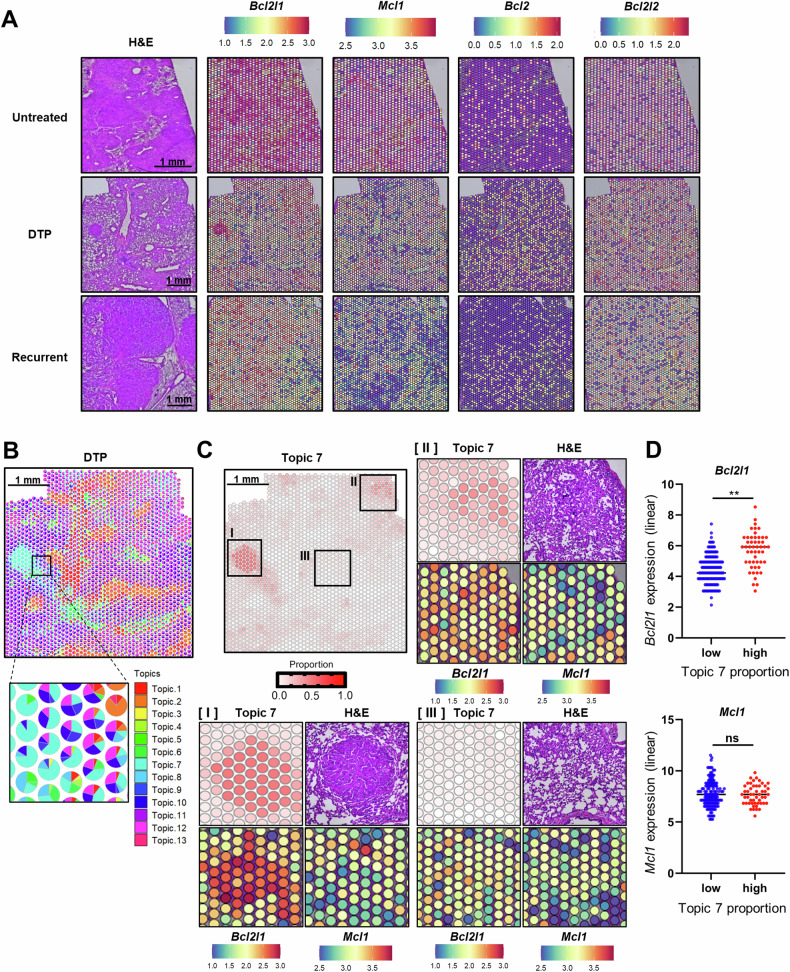
Visualization of inter- and intra-tumor heterogeneity using spatial transcriptomics. **A** H&E staining and *Bcl2l1*, *Mcl1*, *Bcl2, and Bcl2l2* expression in three lung-specific *EGFR-L858R-T790M* (*EGFR*^*TL*^*/CCSP-rtTA*) transgenic mouse samples representing untreated (top), DTP (middle), and tumor-recurrent (bottom) states. **B** Deconvolved cell-type proportions for the DTP dataset, represented as pie charts for each pixel. **C** Pixel proportions showing the distribution of topic 7. Three areas with high (I), medium (II), and low (III) proportions of topic 7 are extracted. Topic 7 proportion, H&E staining, and *Bcl2l1* and *Mcl1* expression levels are visualized in each area. **D** Spots were divided into low (≥25%, <50%) and high (≥50%) groups based on the size of the Topic 7 proportion. *Bcl2l1* and *Mcl1* expression levels were compared between these two groups. *p*-values were calculated using the unpaired two-tailed Welch’s *t*-test. Asterisks indicate *p*-values as follows: **p < 0.005. ns not significant, H&E hematoxylin and eosin, DTP drug-tolerant persister.

Next, to confirm scRNA-seq findings and assess potential inter- and intra-tumor heterogeneity of anti-apoptotic gene expression, we visualized gene expression spatially using Visium analysis of sections from lung-specific *EGFR-L858R-T790M* (*EGFR*^*TL*^*/CCSP-rtTA*) transgenic mice to compare samples that were (1) untreated, (2) treated short-term to represent the DTP state, and (3) treated long-term to represent the recurrent state. Although we observed intra-tumor heterogeneity, *Bcl2l1* expression in tumor lesions was higher than in non-tumor lesions in all states (Fig. 4A), whereas *Mcl1* expression was less tumor-specific and relatively lower in recurrent lesions. Furthermore, *Bcl2* and *Bcl2l2* expression in tumor cells were relatively low and not increased by EGFR-TKI treatment (Fig. 4A). These results overall are consistent with scRNA-seq findings.

Each Visium spot contains multiple cells (spot diameter = 55 µm). Therefore, we used a reference-free deconvolution approach (STdeconvolution) [22] to identify cell types as “Topics” from spot gene expression profiles and assessed their relative proportions in that population. In the DTP state, we identified 13 different cell types (“Topics”) and visualized their relative proportions within a single spot (Fig. 4B, Supplementary Figs. 8 and 9A). The deconvolved transcriptional profile of each “Topic” is shown in Supplementary Table 2 and Supplementary Fig. 9B. Topic 7 was annotated as tumor cells based on that profile, and Topic 7 distribution was consistent with residual tumor lesions, based on hematoxylin and eosin staining (Fig. 4C). We then visualized *Bcl2l1* and *Mcl1* levels based on high (Fig. 4C I), moderate (Fig. 4C II), and low (Fig. 4C III) Topic 7 proportions. Interestingly, *Bcl2l1* expression levels correlated positively with the size of the Topic 7 proportion, while *Mcl1* expression levels did not. Indeed, *Bcl2l1* expression was significantly higher in spots with high (≥50%) Topic 7 proportions than in those with low (≥25%, <50%) Topic 7 proportions. In contrast, no significant difference in *Mcl1* expression was detected between the two groups (Fig. 4D). Taken together, *Bcl2l1* is more highly expressed in residual tumor cells in comparison to neighboring stromal and immune cells.

### BCL-XL loss in lung cancer cells blocks the emergence of drug-tolerant cells and suppresses osimertinib resistance in vitro and in vivo

**Fig. 5:**
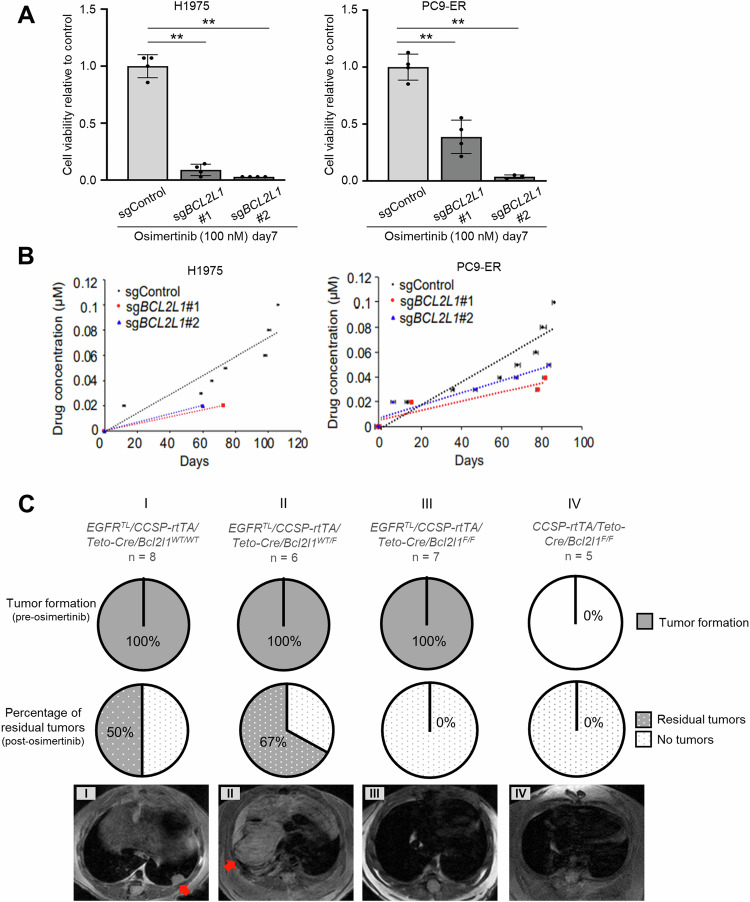
BCL-XL deletion in lung cancer blocks the emergence of drug-tolerant cells and counters osimertinib resistance in vitro and in vivo. **A** The number of cells remaining on day 7 after treatment with 100 nM osimertinib in H1975 (left) and PC9-ER (right) cells transduced with indicated sgRNAs. Data are the mean ± SD of triplicates from one experiment and are representative of three independent experiments. *p*-values were calculated using one-way ANOVA followed by the Tukey–Kramer multiple-comparison test. Asterisks indicate *p*-values as follows: **p < 0.005. **B** Comparison of the time course of acquired osimertinib resistance in H1975 (left) and PC9-ER (right) cells transduced with indicated sgRNAs. Cells were chronically exposed to gradually increasing osimertinib doses. **C** (top) The proportion of indicated transgenic mice exhibiting tumor formation after doxycycline administration; (middle) the percentage of mice exhibiting residual tumors after completion of osimertinib treatment; and (bottom) representative MRI images after completion of osimertinib treatment. Red arrows indicate lung tumors.

Our findings suggest that tumor-specific *BCL2L1* expression likely plays an important role in tumor cell survival, especially in DTP and recurrent states. Thus, we asked whether genetic *BCL2L1* deletion would prevent the emergence of EGFR-TKI-resistant tumors. To do so, we first generated *BCL2L1* knockout (KO) H1975 and PC9-ER cells using CRISPR–CAS9 (Supplementary Fig. 10A) and treated control and *BCL2L1* KO cells for 7 days with osimertinib. After counting surviving cells, we observed significantly fewer cells in *BCL2L1* KO compared to control cells (Fig. 5A). To determine whether *BCL2L1* deletion delayed the emergence of resistant clones, we treated control and *BCL2L1* KO cells with increasing concentrations of osimertinib. Control H1975 and PC9-ER cells showed resistance to 100 nM osimertinib in ~100 and 80 days, respectively. However, *BCL2L1* KO H1975 and PC9-ER cells failed to grow in the medium with 20 nM and 30–40 nM osimertinib, respectively (Fig. 5B).

To confirm these findings in vivo, we crossed *EGFR*^*TL*^*/CCSP-rtTA* mice [23] with conditional *Bcl2l1* knockout mice [24] that had been crossed with tetracycline-inducible Cre-expressing mice (*TetO-Cre/Bcl2l1*^*WT/F*^ or *TetO-Cre/Bcl2l1*^*F/F*^). In resultant *EGFR*^*TL*^*/CCSP-rtTA/TetO-Cre/Bcl2l1*^*WT/WT or WT/F or F/F*^ mice, Cre recombination and induction of EGFR-L858R-T790M occur selectively in pulmonary epithelial cells upon doxycycline treatment (Supplementary Fig. 10B). After doxycycline treatment, we observed tumor formation in all murine lungs, regardless of *Bcl2l1* genotype, suggesting that *Bcl2l1* KO has no effect on tumor formation (Fig. 5C). After induction of tumor formation by doxycycline treatment, we treated mice with osimertinib using a modified intermittent dosing protocol defined in the Methods section [25]. MRI analysis showed that half of the *EGFR*^*TL*^*/CCSP-rtTA/TetO-Cre/Bcl2l1*^*WT/WT*^ mice exhibited resistant tumors, and 4 of 6 mice that carried *EGFR*^*TL*^*/CCSP-rtTA/TetO-Cre/Bcl2l1*^*WT/F*^ exhibited resistant tumors after 4 cycles of treatment (Fig. 5C I and II). In contrast, no *EGFR*^*TL*^*/CCSP-rtTA/TetO-Cre/Bcl2l1*^*F/F*^ mice exhibited resistant tumors (Fig. 5C III). These observations suggest that *BCL2L1*/BCL-XL expression enables tumor cells to resist eradication by EGFR-TKIs.

### Combining osimertinib with BCL-XL inhibitors suppresses osimertinib resistance

**Fig. 6:**
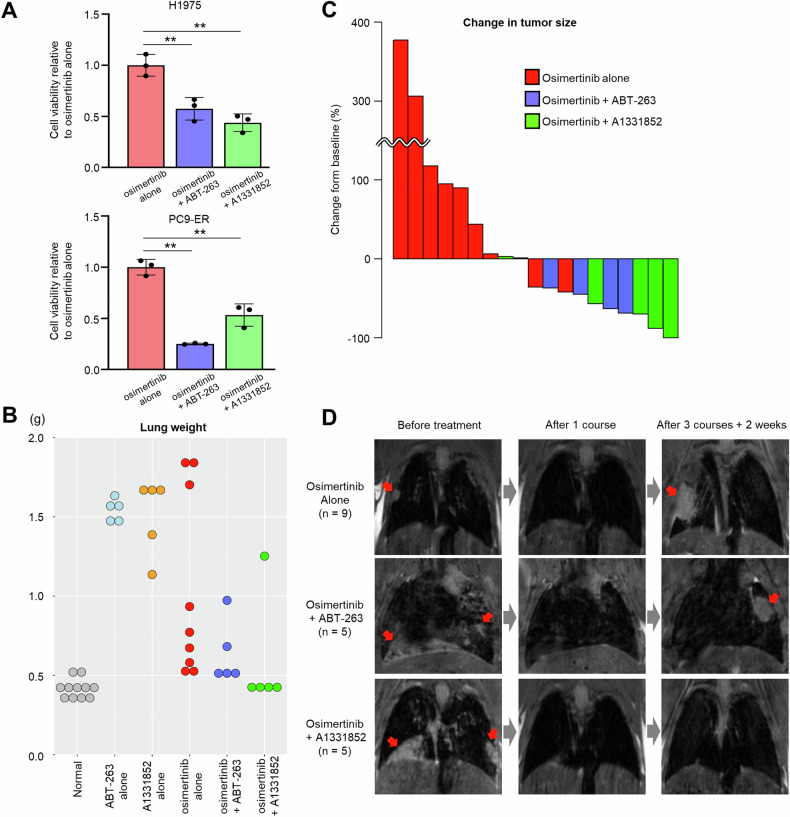
Combining osimertinib with pharmacological BCL-XL inhibition suppresses osimertinib resistance. **A** The number of cells remaining after 7 days of treatment with 100 nM osimertinib plus either 1 µM ABT-263 or 1 µM A1331852 in H1975 (top) and PC9-ER (bottom) cells. Data are the mean ± SD of triplicates from one experiment and are representative of three independent experiments. *p*-values were calculated using one-way ANOVA followed by the Tukey–Kramer multiple-comparison test. Asterisks indicate *p*-values as follows: **p < 0.005. **B** Lung weight at the end of the experiment. Normal lungs were derived from *CCSP-rtTA* transgenic mice (no *EGFR*^*TL*^ expression*)*. **C** Waterfall plot showing changes in tumor size from baseline (before treatment) to the experimental endpoint. The bars shown represent a single mouse. **D** Representative MRI images of mouse lung before and after drug treatment. Red arrows indicate lung tumors. Drug administration protocols are provided in the Methods section.

To determine whether pharmacological BCL-XL inhibition combined with osimertinib treatment blocks drug resistance in *EGFR*-mutated lung cancer, we treated H1975 or PC9-ER cells 7 days with either osimertinib alone or with a combination of osimertinib plus a BCL-XL inhibitor (either ABT-263 or A1331852). Quantification of surviving cells revealed a significant reduction in cell viability following combination therapy with either inhibitor compared to osimertinib alone (Fig. 6A). To determine whether BCL-XL inhibitor treatment delayed the emergence of resistant clones, we treated H1975 cells with a combination of osimertinib plus either of the 2 BCL-XL inhibitors using increasing osimertinib concentrations. Cells treated with osimertinib plus either of the 2 BCL-XL inhibitors required a longer time period to develop resistance to osimertinib compared to cells treated with osimertinib alone (Supplementary Fig. 11).

To confirm these findings in vivo, we treated *EGFR*^*TL*^*/CCSP-rtTA* mice with osimertinib plus either ABT-263 or A1331852, and also with each of the 3 drugs as single agents, and assessed lung weight at the endpoint. The maximum weight of normal lungs obtained from *CCSP-rtTA* transgenic mice (no *EGFR*^*TL*^ expression) was <0.53 g. Notably, we observed lung weights <0.53 g in 2 of 9 (22%) osimertinib-only mice, in 3 of 5 (60%) osimertinib + ABT-263 mice, and in 4 of 5 (80%) osimertinib + A1331852 mice (Fig. 6B). We observed that in groups treated with either ABT-263 or A1331852 alone, all lungs weighed >1.0 g and exhibited massive tumors (Fig. 6B). When comparing tumor sizes post-treatment to their pre-treatment size, only 2 of 9 mice (22%) in the osimertinib-only group showed tumor reduction. Moreover, in groups treated with osimertinib combined with either ABT-263 or A1331852, 4 of 5 mice in each group (80%) exhibited tumor reduction (Fig. 6C). Interestingly, tumor size reduction was greater in the osimertinib + A1331852 combination group. Representative MRI images of this analysis are shown in Fig. 6D.

## Discussion

In this study, we used comprehensive scRNA-seq and ST analysis to identify changes in the expression of apoptosis-related genes after EGFR-TKI treatment and to assess tumor heterogeneity in *EGFR*-mutated lung cancer. We demonstrated that *BCL2L1* is specifically expressed in tumor cells and plays an important role in tumor survival, especially in DTPs and in cells that have acquired resistance to osimertinib treatment.

Apoptosis-related genes reportedly function in tumorigenesis and tumor growth, as well as in drug resistance in some solid tumors [26, 27]. Recently, various DTP cell markers have been reported to function in or mediate EGFR-TKI resistance in *EGFR*-mutated lung cancer, and these could, therefore, serve as novel therapeutic targets [15, 28, 29]. Apoptosis-related genes are known to function in DTP cell formation by acting downstream of these DTP cell marker genes [8, 30]. Our study demonstrated that BCL-XL expression increases at both the mRNA and protein levels following EGFR-TKI treatment and showed that deletion or inhibition of BCL-XL overcame or delayed EGFR-TKI resistance. Interestingly, we observed tumor formation in *BCL2L1* KO mouse lungs, suggesting that *BCL2L1* plays a crucial role in drug resistance but has no impact on tumorigenesis in *EGFR*-mutated lung cancer. Others report that MCL1 activity is important for DTP cell emergence [14]. It is also reported that inhibition of both BCL-XL and MCL1 in cell culture models promotes extensive apoptosis in *EGFR*-mutated lung cancer cells that display an EMT phenotype [31]. However, MCL1 activity in tumor cells varies depending on transcriptional and cellular contexts. In *EGFR*-mutated and *ALK*-rearranged lung cancer cell lines, MCL1 translocates to the nucleus and binds to FBW7 after TKI treatment, which leads to the degradation of MCL1 [32, 33]. MCL1 is also expressed in normal immune cells and is involved in immune cell survival [34]. In the TCGA dataset of lung adenocarcinoma, expression of *MCL1*, *BCL2*, *BCL2L2*, and *BCL2A1* was significantly higher in non-tumor relative to tumor cells (Supplementary Fig. 12). Moreover, our scRNA-seq analysis using human clinical specimens supported the idea that *MCL1* is more highly expressed in non-tumor than in tumor cells, and our ST data also suggests that *Mcl1* expression is not specific to tumors. Interestingly, in a clinical trial against relapsed or refractory hematological malignancies, some MCL1 inhibitors were reported to promote on-target/off-tumor toxicity, and in that trial, administration of the MCL1 inhibitor AMG-397 was suspended due to cardiac side effects [35]. In addition, in a phase I study of the MCL1 inhibitor AMG-176 against relapsed or refractory multiple myeloma, treatment-emergent adverse events of grade 3 or higher occurred in 62% of patients, the most common being neutropenia, anemia, and hypertension [36]. The maximum tolerable AMG-176 dose was not reached, and this trial is also on voluntary hold. Therefore, MCL1 inhibitors are not well-tolerated and are associated with side effects potentially due to effects on normal cells. On the other hand, phase I clinical trials of osimertinib combined with ABT-263 (Navitoclax) for patients with *EGFR*-mutated lung cancer after EGFR-TKI failure have been conducted, and dosing of osimertinib 80 mg daily and navitoclax 150 mg daily was well tolerated [37]. Our study showing that *BCL2L1* expression is higher in tumor cells than in non-tumor cells supports the outcome of this clinical trial. However, combining ABT-263 with osimertinib has not shown striking positive effects for patients with *EGFR*-mutated lung cancer after EGFR-TKI failure. ABT-263 inhibits not only BCL-XL but also BCL-2 and BCL-w. Our scRNA-seq and ST analyses revealed that *BCL2* (BCL-2) and *BCL2L2* (BCL-w) are expressed at low levels in tumor cells and have low tumor specificity (Figs. 3B, E, H, K, N and 4A). Consequently, there may be little benefit to suppressing BCL-2 and BCL-w in this context. Combining A1331852 with osimertinib showed the highest rate of tumor reduction in our in vivo experiments, and selective BCL-XL inhibitors like A1331852 may have more tumor-specific effects, potentially reducing toxicity.

A limitation of our study is the sample size in ST analysis; however, our ST results consistently supported findings obtained from experiments using cell lines and human clinical specimens. Studies of genetic profiles in DTP cells surviving therapeutic assault have thus far relied on cell line-based experiments to define molecular mechanisms underlying DTP cell formation, as ethical constraints preclude the collection of human clinical specimens for this purpose. However, cell line-based experiments do not reflect tumor complexity or diversity or the effects of the tumor microenvironment on DTP cells. We enabled the establishment of a DTP model using *EGFR*-driven lung cancer transgenic mice. This approach allowed for a comprehensive analysis of the dynamics of apoptosis-related genes, including spatial information, during the process of acquiring resistance to EGFR-TKIs. However, further studies are required to understand the mechanism by which DTP cells acquire full drug resistance to EGFR-TKIs and to determine whether our results can be applied to the emergence of resistance to other targeted therapies. Another limitation is that each Visium spot covers multiple cells in our analysis. Therefore, gene expression levels in each spot do not accurately reflect the expression of individual cells, making it challenging to analyze detailed interactions between DTP cells and TME. However, our analysis of ST data showed a positive correlation between tumor cell proportions and *Bcl2l1* expression levels (Fig. 4D), indicating that DTP cells exhibit high *Bcl2l1* expression regardless of co-localized non-tumor cells.

In summary, our multifaceted approach, incorporating new technologies such as scRNA-seq and ST as well as conventional cell line systems and animal models, highlights the importance of *BCL2L1* expression in tumor cell survival as drug resistance emerges and cells acquire resistance to EGFR-TKI.

## Methods

### Cell lines

NCI-H1975 (H1975) cells and HCC827 cells were purchased from ATCC. PC9 cells were kindly provided by Dr. Pasi Jänne. All cell lines were confirmed by short-tandem repeat DNA profiling and routinely tested for mycoplasma using the Mycoalert Mycoplasma Detection Kit (Lonza). PC9-ER cells were established as described [38]. All lines were maintained in RPMI1640 (Corning) plus heat-inactivated 10% FBS (Corning) and 1% penicillin–streptomycin (Corning). Cells were cultured in 5% CO_2_ at 37 °C.

### Generation of stable cell lines harboring sgRNA

pSpCas9-2A-Puro (px459) v2.0 plasmid was obtained from Addgene (Cambridge, MA, USA). DNA Oligos were synthesized and ligated into px459 according to the manufacturer’s protocol. H1975 and PC9-ER lines were transfected with sgRNA plasmids, and stable lines were selected in puromycin to generate *BCL2L1*-knockout (KO) cells. KO efficiency was determined by western blot analysis. Oligo sequences are shown in Supplementary Table 3.

### Western blotting

Cell cytoplasmic extracts were prepared as previously described [15]. SDS-PAGE was performed, and proteins were transferred to PVDF membranes (Millipore Sigma), which were blocked in 5% nonfat milk and PBST and incubated overnight at 4 °C with primary antibodies diluted 1:1000 in 5% BSA. Primary antibodies included those to BCL-XL (Cell Signaling Technology, 2764S) and β-actin (Cell Signaling Technology, 4970S). After incubation with HRP-conjugated secondary antibodies diluted 1:5000 in 5% nonfat milk and PBST, we detected signals with ECL Prime Western Blotting detection reagent (ThermoFisher) using an Amersham imager 600 (GE Healthcare). Expression levels were quantified using Image-J software.

### Quantitative polymerase chain reaction (qPCR)

Total RNA was extracted from cells using an RNA Isolation Kit (ZYMO, Direct-zol RNA Miniprep Plus) and converted to cDNA via RT-PCR using the High-Capacity cDNA Reverse Transcription kit (Thermo Fisher Scientific). Real-time PCR was performed in triplicate using iTaq Universal SYBR Green Supermix (Bio-Rad). Primers are shown in Supplementary Table 4. mRNA abundance was normalized to that of GAPDH.

### Clinical specimens

All patients provided written informed consent before sampling, in accordance with the Declaration of Helsinki. This study was performed in a blinded manner and approved by the National Cancer Center Ethics Committee.

### Single-cell RNA-seq analysis

scRNA-seq data acquisition and analysis workflow were previously described [15]. Briefly, scRNA-seq analysis was performed using R (v4.2.1). Using Seurat ver4.3.0 [39], low-quality reads and PCR “sister” duplicates were removed. To filter out low-quality cell data, the following threshold was determined for each sample. For cell lines, (i) >7500 UMI per cell, (ii) >1000 genes per cell, and (iii) <10% mitochondrial gene expression; for Pt-1 and -3, (i) >5000 UMI per cell, (ii) >1000 genes per cell, and (iii) <20% mitochondrial gene expression; for Pt-2, -4 and -5, (i) >5000 UMI per cell, (ii) >1000 genes per cell, and (iii) <10% mitochondrial gene expression. Stats of the scRNA-seq analysis are shown in Supplementary Table 1.

### Transgenic murine models

All animal studies were approved by the Institutional Animal Care and Use Committee at Beth Israel Deaconess Medical Center. *EGFR-L858R-T790M* (*EGFR*^*TL*^*)/CCSP-rtTA* bi-transgenic mice and *teto-Cre* transgenic mice were previously described [23, 40]. *Bcl2l1* floxed mice were provided by Dr. Lothar Hennighausen at the National Institute of Diabetes and Digestive and Kidney Diseases. Samples were collected until the sample size was sufficient to give comparison and reliable estimates. Once male and female juvenile mice reached three to five weeks of age, they were treated with doxycycline for eight to ten weeks to induce *EGFR*^*TL*^ expression and excise *Bcl2l1*. Immediately after doxycycline treatment, mice underwent magnetic resonance imaging (MRI). For in vivo studies, we modified a protocol previously published to establish an erlotinib-resistant mouse model [25]. In brief, osimertinib at 5 mg kg^−1^ per day, ABT-263 (Chemgood, C-1009) at 100 mg kg^−^^1^ per day, and A1331852 (MedChemExpress, HY-19741) at 25 mg kg^−1^ per day were administered by oral gavage 6 days per week with drug-on/drug-off cycles described below, while maintaining mice on a doxycycline-containing diet: (i) mice for ST and for the pharmacological BCL-XL inhibition study, 2 weeks on/2 weeks off for 2 courses, then 4 weeks on; (ii) *Bcl2l1* knockout evaluation study, 4 weeks on/2 weeks off for 4 courses, then 2 weeks on. Animals were randomly grouped for the pharmacological BCL-XL inhibition study. No randomization was required in the other study using mouse models since no treatment conditions were compared. The investigators were blinded to group allocation during data analysis. Tumor growth was monitored by MRI. The sum of maximum tumor diameters was calculated using Sante DICOM Viewer Lite 3.2.4. Response criteria were defined as follows: partial response (PR), at least a 30% decrease in the sum of diameters of target lesions, taking baseline sum diameters as a reference; progressive disease (PD), at least a 20% increase in the sum of diameters of target lesions, taking the smallest sum in the study as a reference; and stable disease (SD), neither sufficient shrinkage to qualify for a PR nor sufficient increase in tumor size to qualify for PD. Mouse lungs were dissected and subjected to histological analysis. Tissues were embedded in paraffin, sectioned, and stained with hematoxylin and eosin in the Histology Core Facility at Beth Israel Deaconess Medical Center.

### Library preparation and data pre-processing of Spatial Transcriptomics (ST)

ST experiments were performed using the 10× Visium Spatial Gene Expression kit. Protocols for Tissue Optimization and Library preparation were followed based on the manufacturer’s instructions. Library preparation data pre-processing and normalization were done in the Spatial Technology Unit at Beth Israel Deaconess Medical Center. In brief, a 5 μm-thick section was prepared from the FFPE blocks and processed using the Visium Spatial Gene Expression for FFPE Kit (10× Genomics) according to the manufacturer’s instructions. Sections were H&E stained and imaged, followed by probe hybridization and ligation. Libraries were sequenced to a depth of more than 25,000 read pairs per spot for Visium experiments. Reads were processed using Spaceranger v2.0.0 with mm10 (build 2020-A, 10× Genomics) and Visium Mouse Transcriptome Probe Set v1.0 as references. For Visium spatial transcriptomics data, tissue morphology was annotated at per spot level using the Loupe browser (v6.2.0, 10× Genomics) and STUtility v1.1.1 [41]. Spots underneath regions affected by processing artifacts were annotated as “exclude” and excluded from downstream analyses. The UMI counts were then normalized using the SCTransform (v0.3.5) function from the Seurat package with R (v4.2.1).

### Cell-type deconvolution

To infer the spatial organization of certain cell types in Day 4 samples, we used a reference-free deconvolution approach using the STdeconvolve (ver 1.2.0) R package [22]. Briefly, to first select genes in the LDA model, genes detected in either <5% or in 100% of pixels were removed. We optimize the number of cell types *K* in the LDA model to minimize perplexity and the number of predicted cell-types with mean pixel proportion less than 5%, and then determined the number of cell types *K* in the LDA model to be 13.

### Annotating deconvolved cell-types

Deconvolved cell types (topics) were annotated based on PanglaoDB [42], a database with single-cell datasets and lists of cell-type markers for various human and mouse tissues. Annotated cell types were subjected to functional enrichment analysis using g:profiler [43]. Each cell type was then manually annotated on the basis of top-enriched pathways and marker gene upregulation.

### Statistics

Statistical analyses were performed with GraphPad Prism 9.2.0. Two-group means comparison was analyzed using the unpaired two-tailed Welch’s *t* test. To evaluate multigroup data sets, one-way ANOVA with a Tukey–Kramer multiple-comparison test was used for the parametric test, and the Kruskal–Wallis one-way ANOVA was used for the non-parametric comparison. Also, a Repeated Measures one-way ANOVA with a Dunnett’s test was used to compare a treatment group to a single control group. *p*-values < 0.05 were considered statistically significant. Data are presented as mean ± SD.

### Supplementary information

Supplementary Figure 1-12 and Table 1, 3, 4

Supplementary Table 2

Uncropped western blots

## Data Availability

Datasets generated or analyzed during the current study are available from the corresponding author on reasonable request. The public single-cell sequencing dataset applied in this study is on the GEO database with number GSE146100.

## Code availability

The version and parameters for the R packages used in this study are available in the Material and Methods section. Any additional information required to reanalyze the data reported in this study is available from the corresponding author on reasonable request.
