# Supplementary material for: Integrative single-cell RNA-seq and spatial transcriptomics analyses reveal diverse apoptosis-related gene expression profiles in EGFR-mutated lung cancer
Source: Cell Death Dis. 2024 Aug 9;15(8):580. doi: 10.1038/s41419-024-06940-y (PMC11316060; doi:10.1038/s41419-024-06940-y)
Supplement: Supplementary file 1 — Supplementary Figure 1-12 and Table 1, 3, 4 [file 41419_2024_6940_MOESM1_ESM.pdf]

Supplementary Information

Integrative single-cell RNA-seq and spatial transcriptomics analyses reveal diverse apoptosis-related gene expression profiles in *EGFR*-mutated lung cancer

Authors

Motohiro Izumi[1, 5], Masanori Fujii[1, 5], Ikei S. Kobayashi[1], Vivian Ho[1], Yukie Kashima[2], Hibiki Udagawa[2, 3], Daniel B Costa[1], Susumu S. Kobayashi[1, 2, 4*]

Affiliations

[1]Department of Medicine, Division of Medical Oncology, Beth Israel Deaconess Medical Center, Harvard Medical School, Boston, MA, 02215, USA.

[2]Division of Translational Genomics, Exploratory Oncology Research and Clinical Trial Center, National Cancer Center, Kashiwa 277-8577, Japan.

[3]Department of Thoracic Oncology, National Cancer Center Hospital East, Kashiwa 277-8577, Japan.

[4]Department of Respiratory Medicine, Juntendo University Faculty of Medicine and Graduate School of Medicine, Tokyo, 113-8431, Japan.

[5]These authors contributed equally to this work.

[*]Correspondence to: skobayas@bidmc.harvard.edu

Supplementary Fig. 1

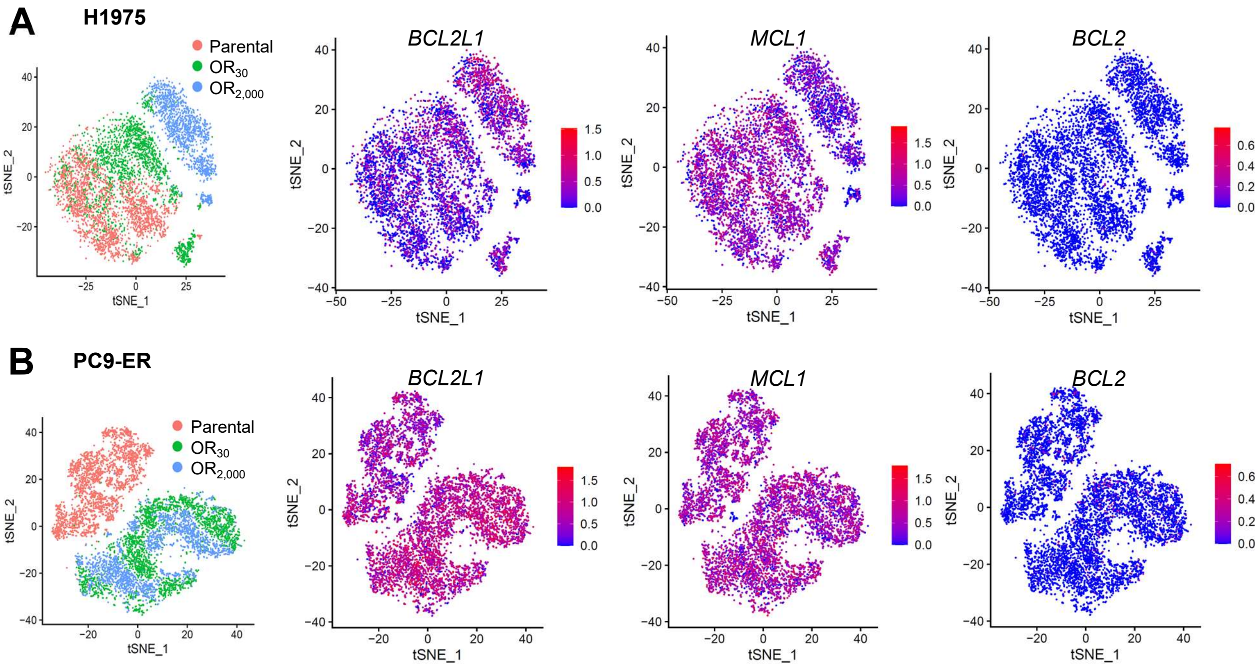

Supplementary Fig. 1. scRNA-seq analysis of osimertinib-resistant lung cancer line models.
A, B t-SNE plots showing *BCL2L1* (left), *MCL1* (middle), and *BCL2* (right) expression in H1975 (A) and PC9-ER (B) datasets.

Supplementary Fig. 2

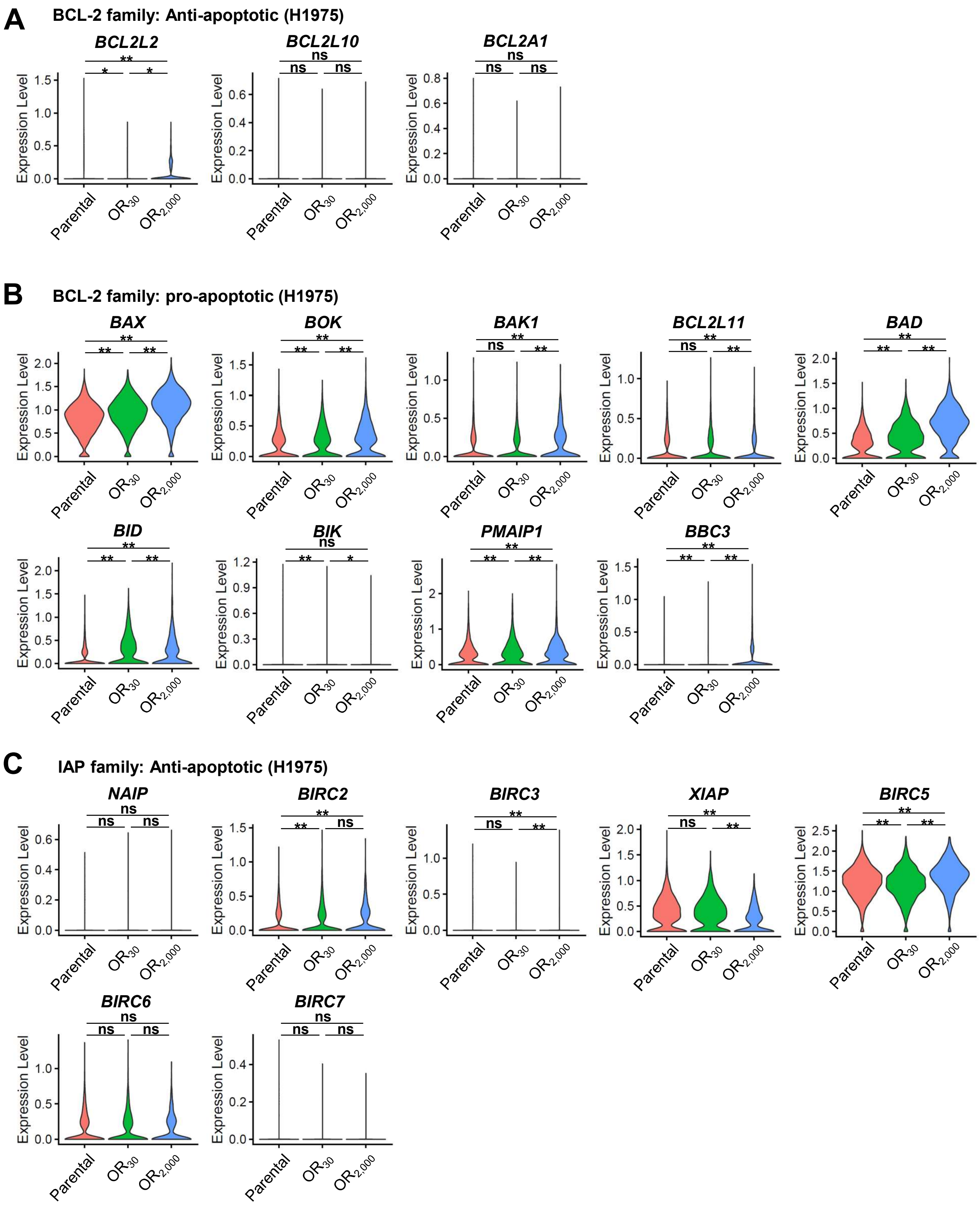

Supplementary Fig. 2. Comprehensive apoptosis-related gene expression in H1975 cells based on scRNA-seq. A, B Violin plots showing expression of (A) anti-apoptotic and (B) pro-apoptotic BCL-2 family genes in parental, OR_{30}, and $OR_{2,000}$ H1975 cell lines. **C** Comparable Violin plots showing expression of anti-apoptotic IAP family genes. p-values were calculated using Kruskal–Wallis one-way ANOVA. Asterisks indicate p-values as follows: *$p < 0.05$; **$p < 0.005$; ns, not significant.

Supplementary Fig. 3

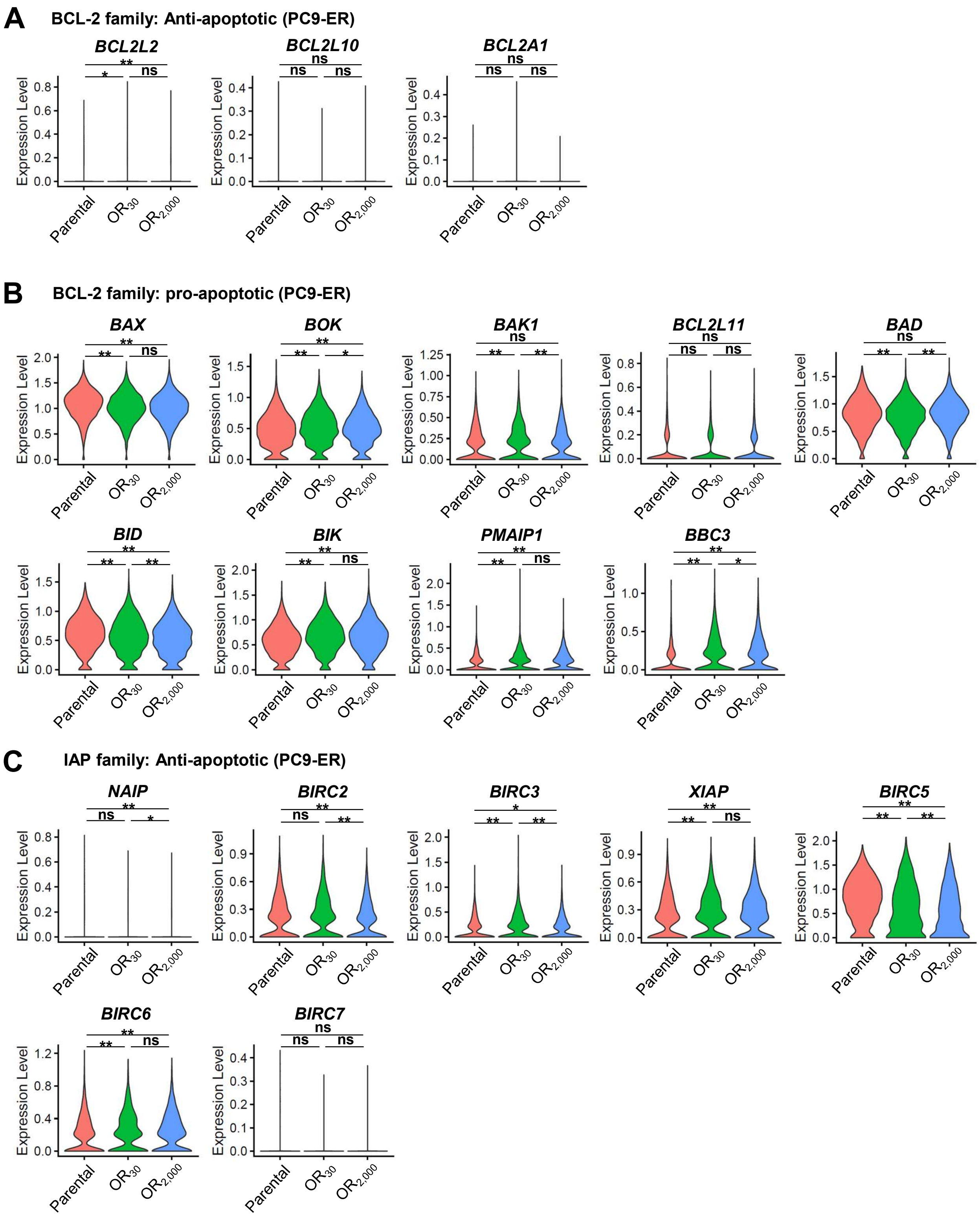

Supplementary Fig. 3. Comprehensive apoptosis-related gene expression in PC9-ER cells based on scRNA-seq. A, B Violin plots showing expression of (A) anti-apoptotic and (B) pro-apoptotic BCL-2 family genes in parental, OR_{30}, and $OR_{2,000}$ in PC9-ER cell lines. **C** Comparable Violin plots showing expression of anti-apoptotic IAP family genes. p-values were calculated using Kruskal–Wallis one-way ANOVA. Asterisks indicate p-values as follows: *$p < 0.05$; **$p < 0.005$; ns, not significant.

Supplementary Fig. 4

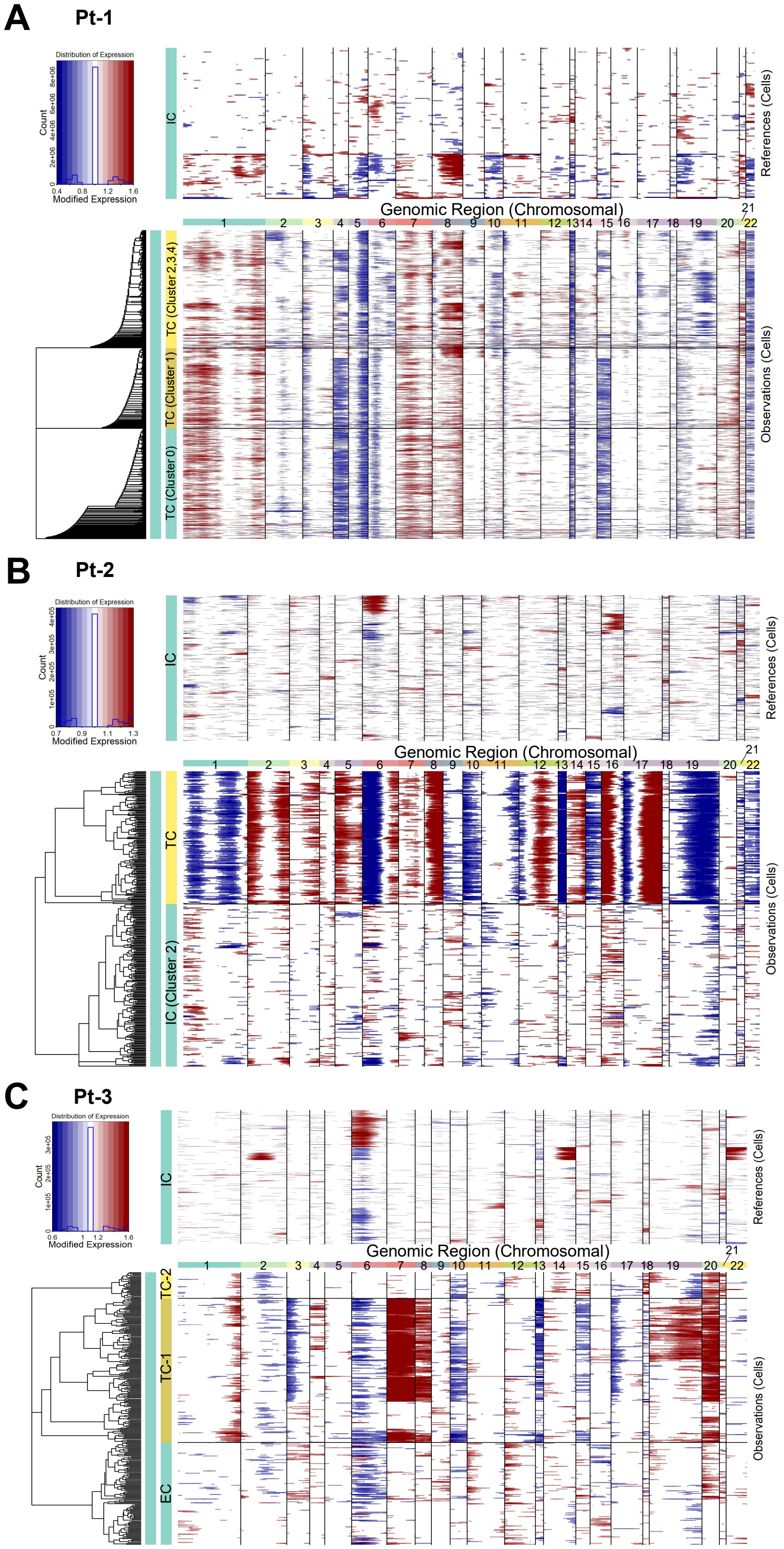

Supplementary Fig. 4. Copy number variants in Pt-1, Pt-2, and Pt-3 patient specimens. A, B, C Inferred CNV heatmaps showing somatic large-scale chromosomal copy number alterations, such as gains or deletions, in tumor cells compared to non-tumor cells derived from indicated patients. *TC,* tumor cell; *IC,* immune cell; *EC,* endothelial cell.

Supplementary Fig. 5

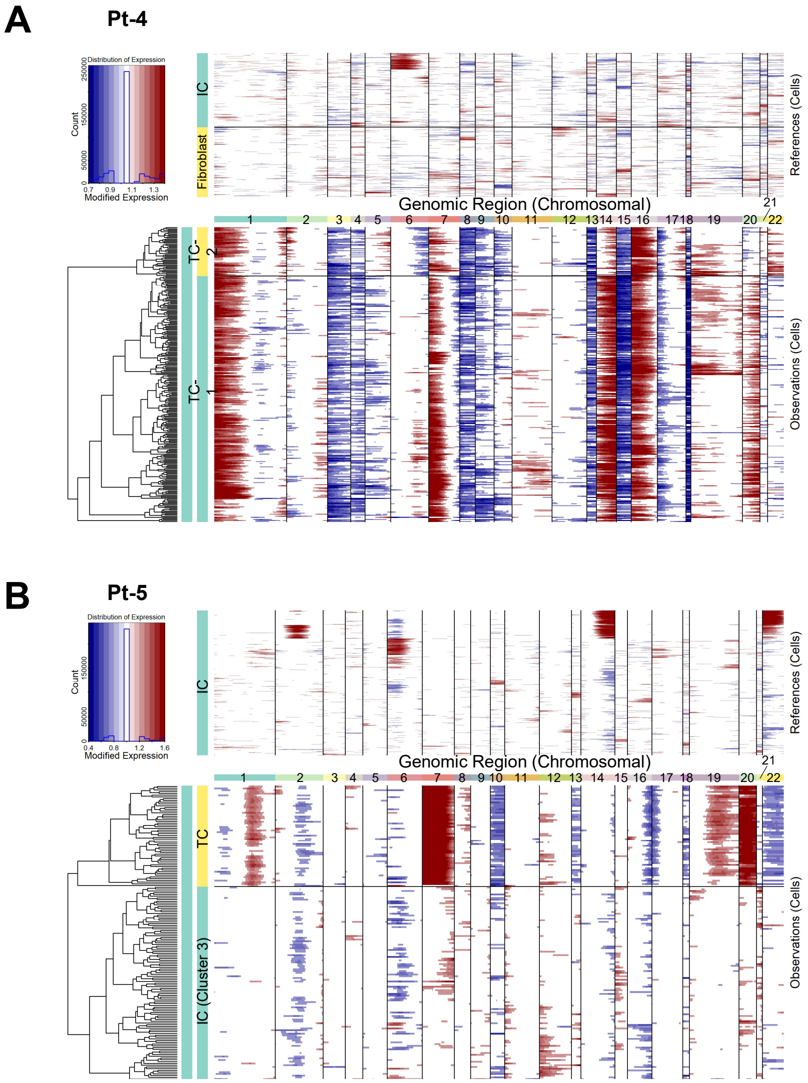

Supplementary Fig. 5. Copy number variants in patient specimens Pt-4 and Pt-5. A, B Inferred CNV heatmaps showing somatic large-scale chromosomal copy number alterations, such as gains or deletions, in tumor cells compared to non-tumor cells derived from indicated patients. *TC,* tumor cell; *IC,* immune cell.

Supplementary Fig. 6

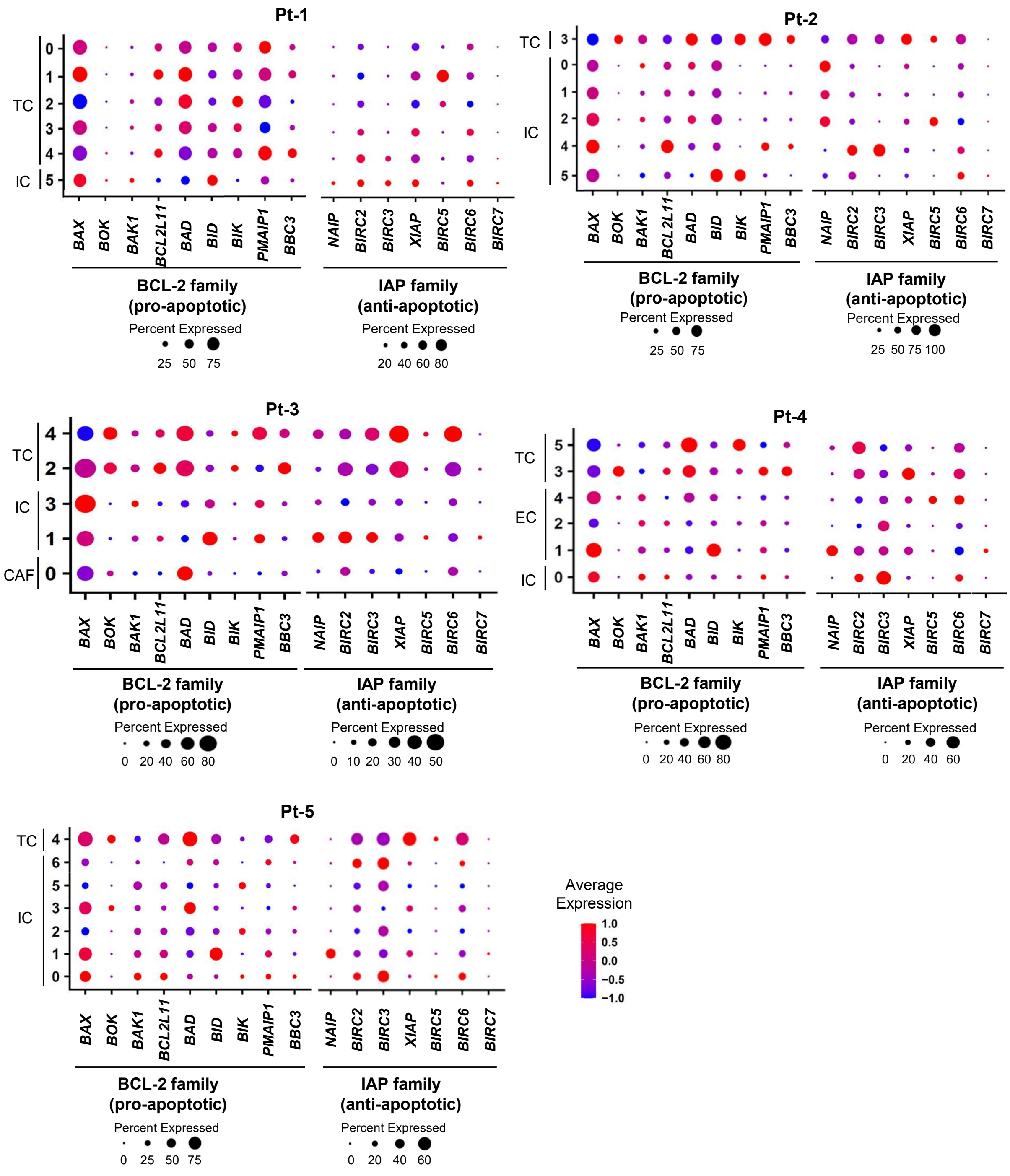

Supplementary Fig. 6. Expression of pro-apoptotic BCL-2 family genes and anti-apoptotic IAP family genes in clinical samples. Dot plots showing differences in apoptosis-related gene expression between tumor and non-tumor cells. *TC,* tumor cell; *IC,* immune cell; *EC,* endothelial cell; *CAF*, cancer-associated fibroblast.

Supplementary Fig. 7

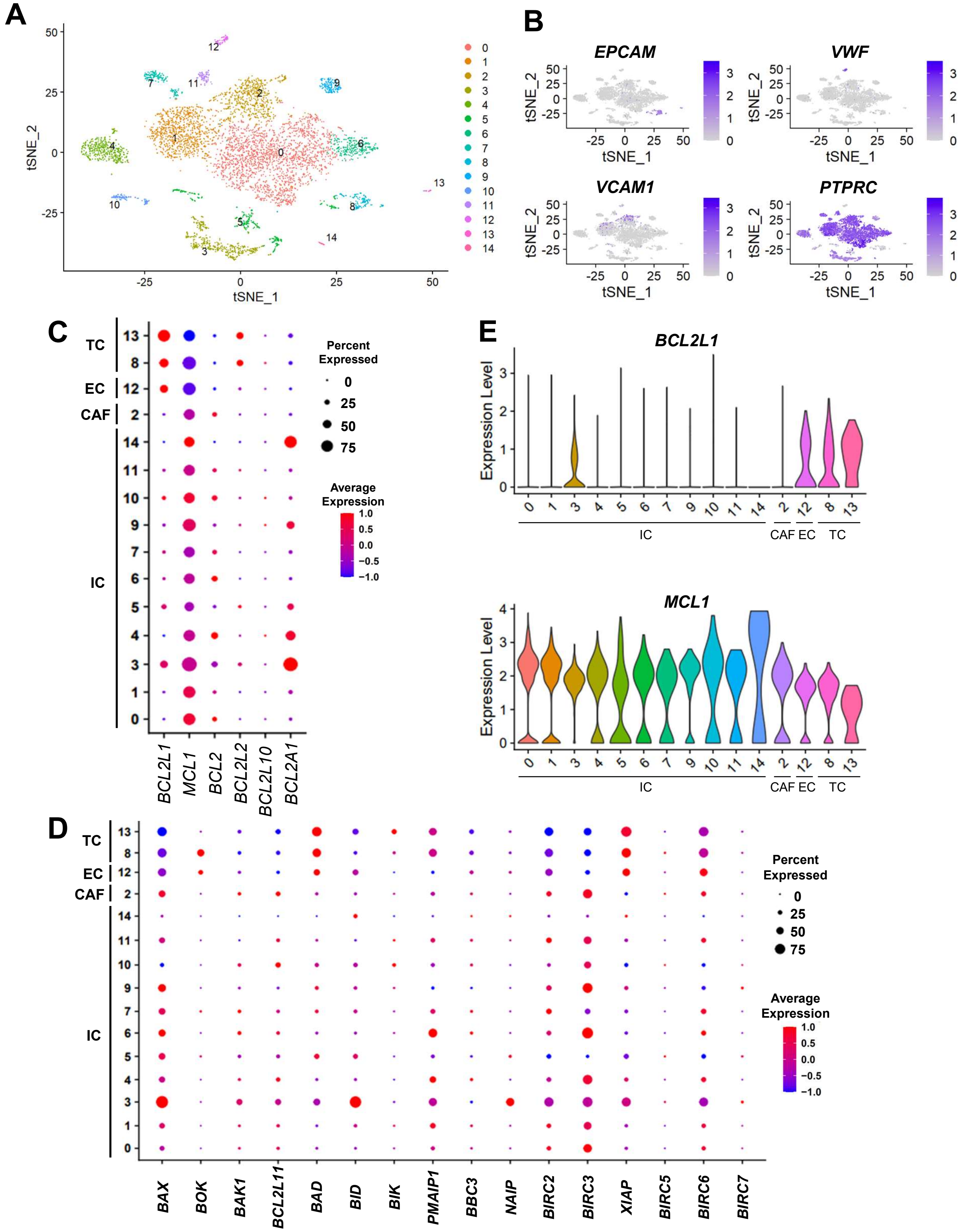

Supplementary Fig. 7. Expression apoptosis-related genes in a clinical sample (GSE146100). A t-SNE plots showing clusters based on scRNA-seq. **B** t-SNE plots showing expression of cell type-specific marker genes (*EPCAM*, tumor cells; *VWF*, epithelial cells; *VCAM1*, CAF; and *PTPTRC*, CD45-positive immune cells). **C, D** Dot plots showing differences in apoptosis-related gene expression between tumor and non-tumor cells. **E** Violin plots showing *BCL2L1* (upper) and *MCL1* (lower) expression in indicated cell types. *TC,* tumor cell; *IC,* immune cell; *EC,* endothelial cell; *CAF*, cancer-associated fibroblast.

Supplementary Fig. 8

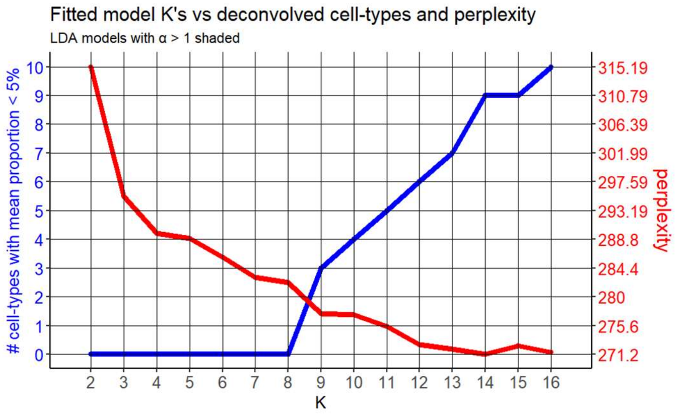

Supplementary Fig. 8. STdeconvolve *K* versus cell-types with mean proportion less than 5% and perplexity. Perplexity and the number of predicted cell types with mean pixel proportion less than 5% in each the number of cell types *K*.

Supplementary Fig. 9

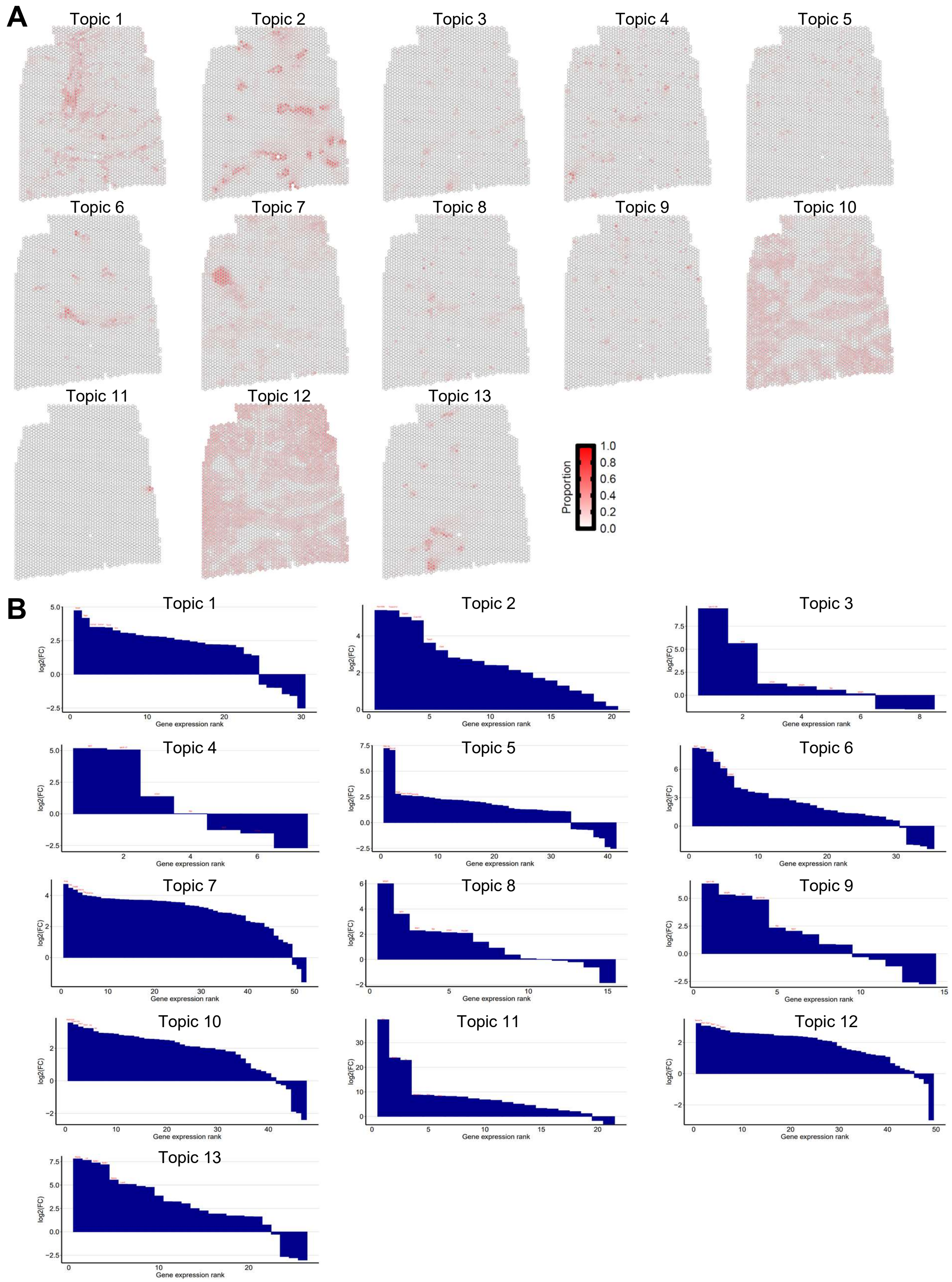

Supplementary Fig. 9. Spatial transcriptomics analyses of *EGFR*-driven lung cancer transgenic mice.

A Distribution of each deconvolved cell-type (Topic type). **B** Differentially-expressed genes for each deconvolved cell-type transcriptional profile.

Supplementary Fig. 10

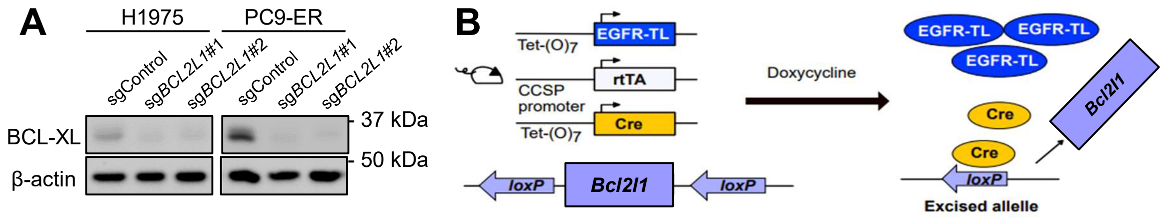

Supplementary Fig. 10. Generation of *BCL2L1* KO models. A Western blot confirming establishment of H1975 (left) and PC9-ER (right) cell lines stably expressing sgRNA targeting *BCL2L1,* which encodes BCL-XL. **B** Scheme showing constructs (*TetO-EGFR-L858R-T790M (EGFR*^{TL}*)*, *CCSP-rtTA*, and *TetO-Cre*) used to generate *EGFR*-mutated lung cancer mouse models with doxycycline-inducible, lung-specific conditional *Bcl2l1* deletion in pulmonary alveolar cells.

Supplementary Fig. 11

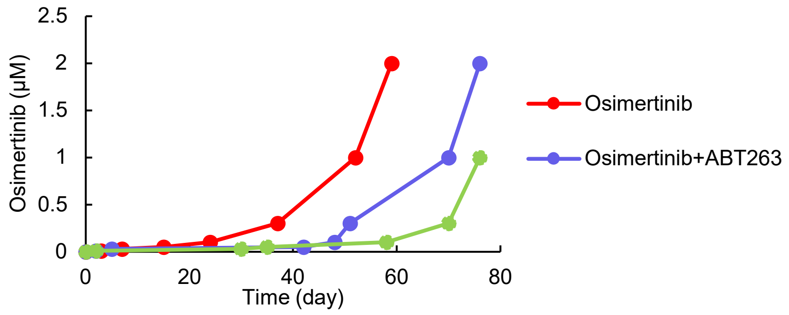

Supplementary Fig. 11. ABT-263 and A1331852 delay the emergence of osimertinib resistance
H1975 cells were treated with gradually increasing concentrations of osimertinib either alone, or combined with ABT-263 (1μM) or A1331852 (1μM) and the days required to increase the concentration of osimertinib were plotted until developing resistance to 2 μM osimertinib.

Supplementary Fig. 12

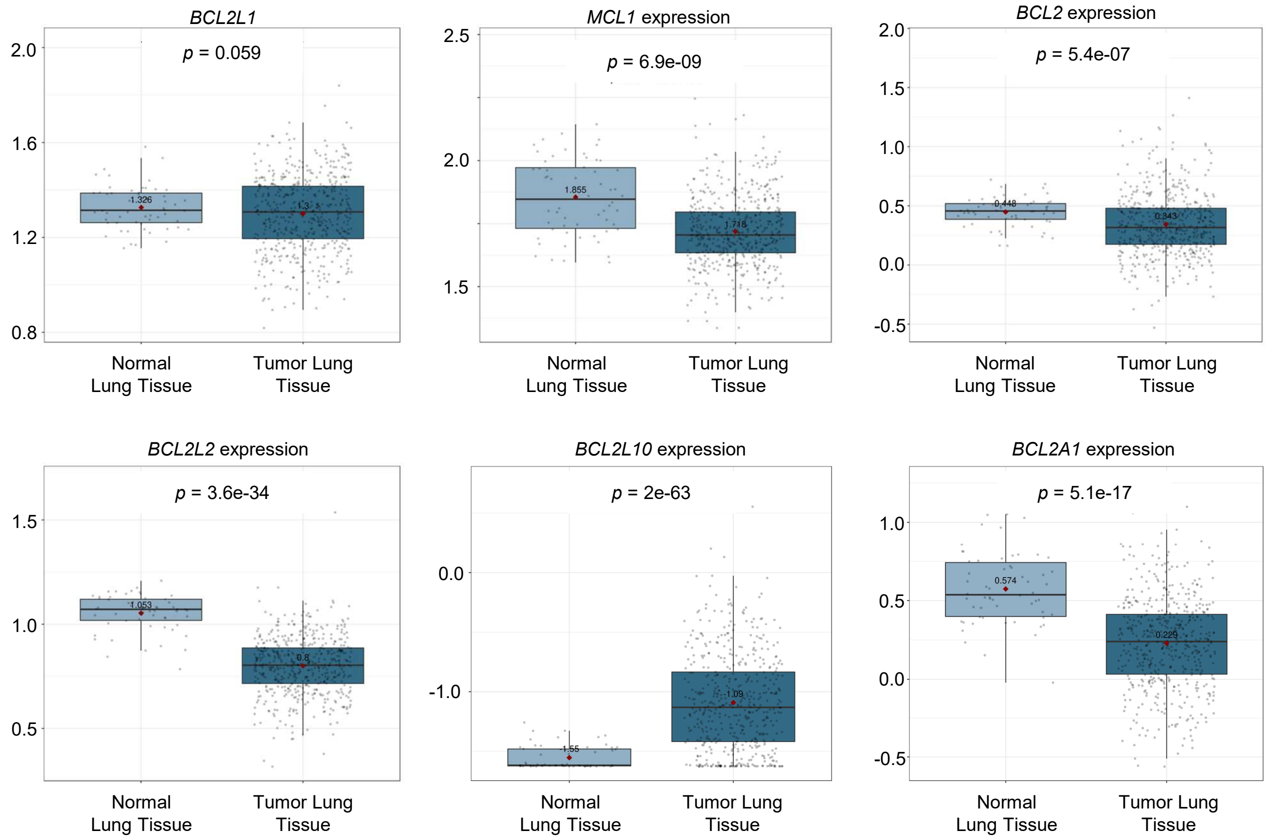

Supplementary Fig. 12. Comparison of expression levels of anti-apoptotic genes in normal versus tumor lung tissue in the TCGA dataset of lung adenocarcinoma. Comparative analysis showing that expression of indicated anti-apoptotic BCL2 family genes is significantly higher in non-tumor (n = 58) versus tumor (n = 517) cells, with the exception of *BCL2L1* and *BCL2L10*. Boxes show medians with upper and lower quartiles, and whiskers represent minimum and maximum values. For comparative analysis, p-values were calculated using the unpaired two-tailed Welch's t-test.

Supplementary Tables

Supplementary Table 1. Patient characteristics

Characteristic	Pt-5
Histology	Adenocarcinoma
EGFR Mutations	EGFR exon19del
Sex	M
Age	76
Smoking	92 pack-years
Treatments	de novo
Sample types	Bronchoscopy biopsy
Raw reads	319,860,004
total cells after cut-off	569
included tumor cells	62

Supplementary Table 3. Oligos used to establish stable knockout cell lines of indicated gene

Gene	Oligos	5' - 3'
BCL2L1	#1	GCAGACAGCCCCGCGGTGAA
	#2	CCATCAATGGCAACCCATCC

Supplementary Table 4. Primers used for quantitative PCR analysis

Gene	Primers	5' - 3'
BCL2L1	F	TGAGTGAGCAGGTGTTTTGG
	R	GGATCCAAGGCTCTAGGTGG
GAPDH	F	CCAGGCGCCCAATACG
	R	CCACATCGCTCAGACACCAT
