# Supplementary material for: Integrative single-cell RNA-seq and spatial transcriptomics analyses reveal diverse apoptosis-related gene expression profiles in EGFR-mutated lung cancer
Source: Cell Death Dis. 2024 Aug 9;15(8):580. doi: 10.1038/s41419-024-06940-y (PMC11316060; doi:10.1038/s41419-024-06940-y)

Supplementary Information

Uncropped scan images used in this manuscript. Cropped imaged used are indicated by red squares.

Fig. 2D

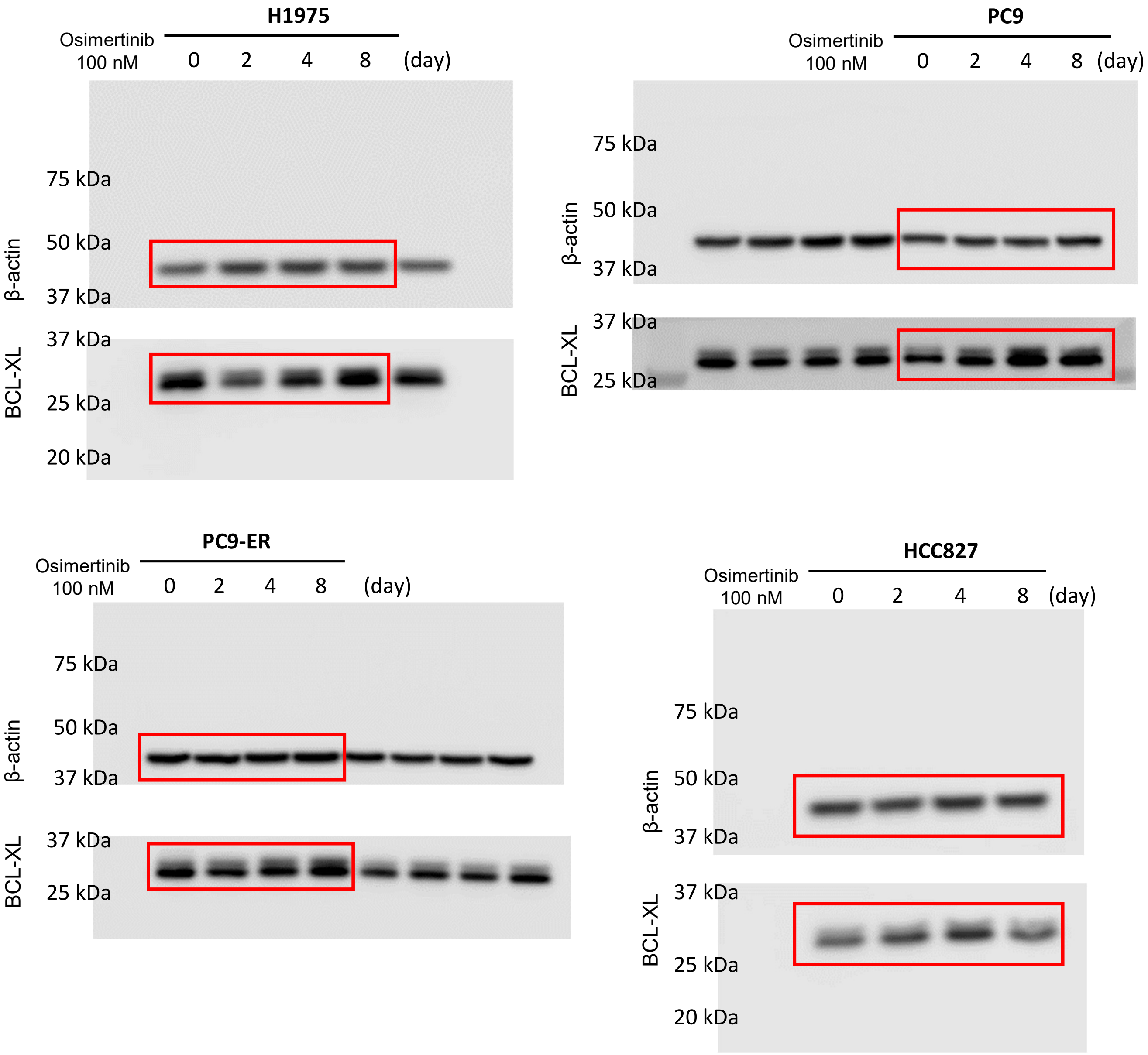

Supplementary Fig. S10A

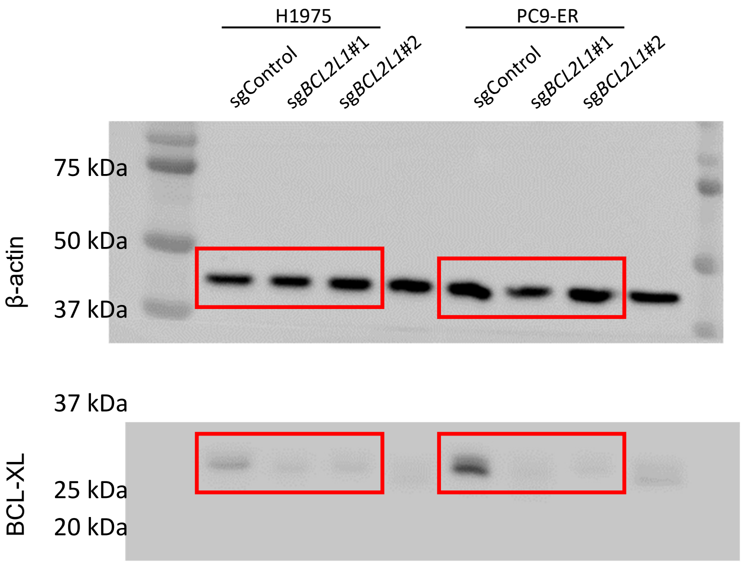

Supplement: Supplementary file 3 — Uncropped western blots [file 41419_2024_6940_MOESM3_ESM.pdf]
